# Prostaglandins induce early growth response 1 transcription factor mediated microsomal prostaglandin E_2_ synthase up-regulation for colorectal cancer progression

**DOI:** 10.18632/oncotarget.5402

**Published:** 2015-10-17

**Authors:** Konstantinos Stamatakis, Marta Jimenez-Martinez, Alba Jimenez-Segovia, Isabel Chico-Calero, Elisa Conde, Javier Galán-Martínez, Julia Ruiz, Alejandro Pascual, Beatriz Barrocal, Ricardo López-Pérez, María Laura García-Bermejo, Manuel Fresno

**Affiliations:** ^1^ Centro de Biología Molecular ‘‘Severo Ochoa” (Consejo Superior de Investigaciones Científicas-Universidad Autónoma de Madrid), Universidad Autónoma de Madrid, Madrid, Spain; ^2^ Instituto Ramón y Cajal de Investigación Sanitaria (IRYCIS), Carretera de Colmenar, Madrid, Spain; ^3^ Instituto Sanitario Princesa de Investigacion Sanitaria (IIS-P), Madrid, Spain

**Keywords:** cyclooxygenase 2, microsomal prostaglandin E_2_ synthase, colorectal adenocarcinoma, early growth response 1

## Abstract

Cyclooxygenase2 (COX2) has been associated with cell growth, invasiveness, tumor progression and metastasis of colorectal carcinomas. However, the downstream prostaglandin (PG)-PG receptor pathway involved in these effects is poorly characterized.

We studied the PG-pathway in gene expression databases and we found that *PTGS2* (prostaglandin G/H synthase and cyclooxygenase) and *PTGES* (prostaglandin E synthase) are co-expressed in human colorectal tumors. Moreover, we detected that COX2 and microsomal Prostaglandin E_2_ synthase 1 (mPGES1) proteins are both up-regulated in colorectal human tumor biopsies.

Using colon carcinoma cell cultures we found that COX2 overexpression significantly increased mPGES1 mRNA and protein. This up-regulation was due to an increase in early growth response 1 (EGR1) levels and its transcriptional activity. EGR1 was induced by COX2-generated PGF_2α_. A PGF_2α_ receptor antagonist, or *EGR1* silencing, inhibited the mPGES1 induction by COX2 overexpression. Moreover, using immunodeficient mice, we also demonstrated that both COX2- and mPGES1-overexpressing carcinoma cells were more efficient forming tumors.

Our results describe for the first time the molecular pathway correlating *PTGS2* and *PTGES* in colon cancer progression. We demonstrated that in this pathway mPGES1 is induced by COX2 overexpression, via autocrine PGs release, likely PGF_2α_, through an *EGR1*-dependent mechanism. This signaling provides a molecular explanation to *PTGS2* and *PTGES* association and contribute to colon cancer advance, pointing out novel potential therapeutic targets in this oncological context.

## INTRODUCTION

Colorectal cancer is the most common malignancy and the second most common cause of cancer death in Europe [1]. Even in patients who have undergone tumor resection, 40–50% relapse and die of metastases, being the overall 5-year survival less than 60% [2]. Thus, the currently available treatments do not achieve the desired efficiency and development of new therapeutic strategies is required.

Clinical trials and epidemiological studies have suggested that cyclooxygenase 2 (COX2) is involved in colorectal cancer development and its inhibition can reduce the risk of cancer in general and more specifically of colorectal cancer [3–7].

The activity of cyclooxygenases (COX) is coupled to several terminal synthases that produce the different PGs [8]. The major prostaglandins produced are the PGE_2_, PGD_2_, PGF_2α_, PGI_2_ by their respective synthases and are present in the healthy colon as well as in colorectal cancer [9]. PGE_2_ has been proposed as the principal prostanoid associated to colorectal tumors since PGE_2_ levels are elevated in patients with colon cancer and correlate with tumor size [10]. Three PGE_2_ synthases have been described [11], two microsomal, mPGES1, mPGES-2 and the cytoplasmic cPGES, encoded by the *PTGES*, *PTGES2* and *PTGES3* genes, respectively. mPGES1 expression has been associated to colorectal cancer incidence and prognosis [12, 13] and has been proposed to cooperate with COX2 to enhance tumor growth [14].

Early growth response 1 (*EGR1*) is a transcriptional regulator that belongs to the EGR family of the C2H2-type zinc finger proteins. *EGR1* is rapidly induced by a variety of stimuli, such as lipopolysaccharide, cytokines and growth factors, and regulates gene expression of proteins required for mitosis, inflammatory responses and differentiation [15]. It has been established that several prostanoids and more particularly PGF_2α_ and PGE_2_ can induce *EGR1* expression [16, 17]. *EGR1* can also activate mPGES1 expression coordinately with NFκB or by itself in a variety of cell types [18–20]. Finally, *EGR1* has been shown to induce apoptosis in cancer cells when up-regulated by NSAIDs [21, 22], thus the role of EGR1 is controversial.

In contrast to the wealth of data relating COX2 and colon cancer, there is little direct experimental evidence demonstrating that the expression/activity of COX2 or mPGES1 is causally linked to tumor progression and metastasis, in colorectal cancer. Besides, the PGs pathways downstream of COX2 mediating its pro-tumoral effects are mostly unknown. Thus, to further investigate the direct effects of the COX2/prostanoids pathway by itself in colorectal tumor progression, we generated stable colon carcinoma cell lines that overexpressed the human COX2 gene. These cell lines have increased tumorigenic capacity both *in vitro* and *in vivo* and we found altered expression levels of various components of the PG pathway including mPGES1. Notably, we found co-localization of COX2 and mPGES1 in human colorectal cancer biopsies and human tumor array data sets. Besides, overexpression of mPGES1 was enough to provide cells with an increased tumorigenic capacity in immunodeficient mouse xenograft models. This is the first report to demonstrate that high levels of any of the two enzymes are sufficient to enhance colorectal tumor growth and that COX2 activity induces mPGES1 expression through *EGR1*.

## RESULTS

### PGs pathway in colorectal cancer tumors

**Figure 1:**
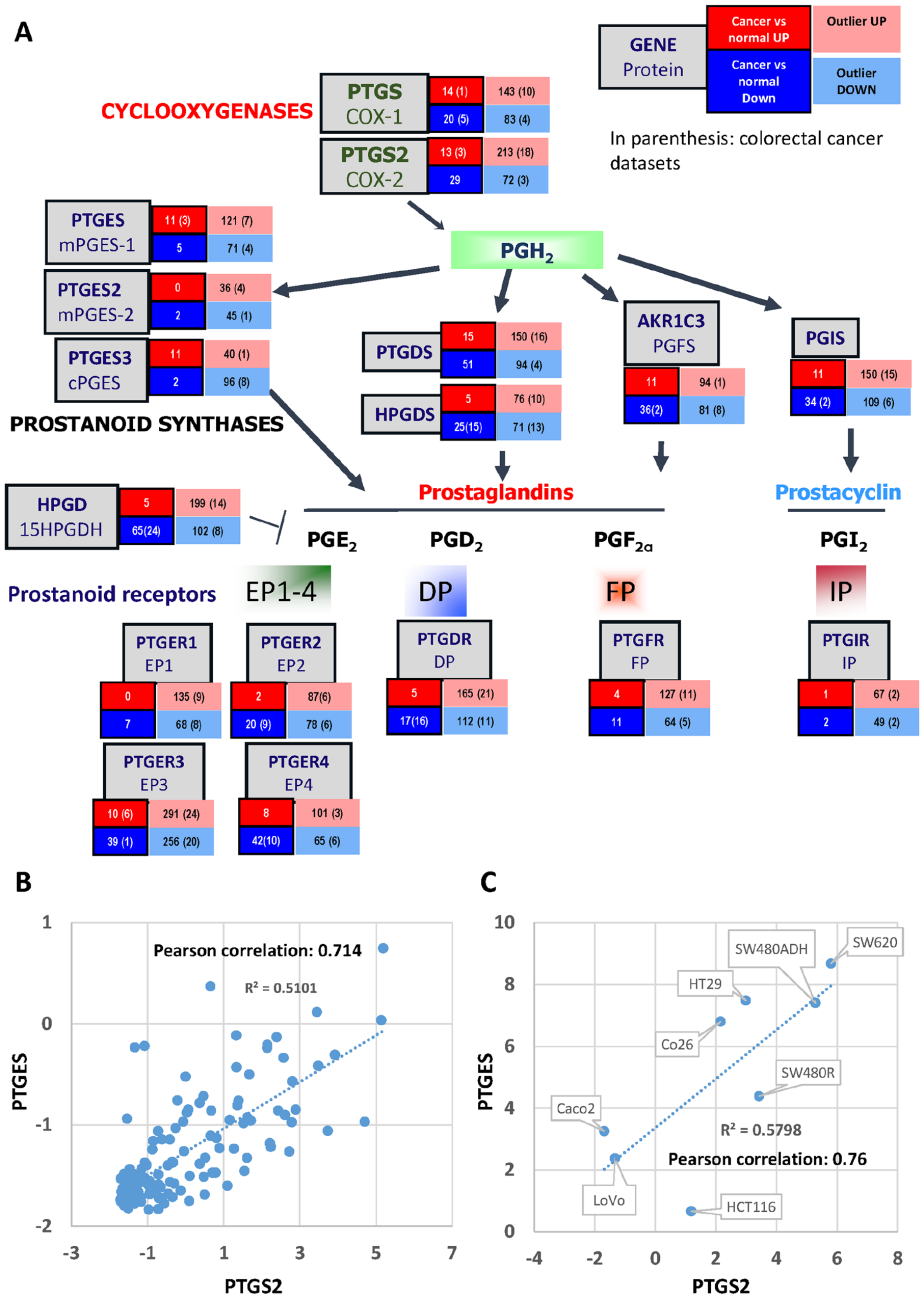
PG Partway gene expression analysis in human tumors **A.** Summary from the data obtained from the “Gene summary view” of the Oncomine database (Oncomine 2014, Compendia Bioscience) for each of the genes. The number of tumor collections where each gene was found significantly up- or down- regulated in tumor vs normal analyses is shown in the red and blue boxes respectively. In the pink and light-blue boxes are shown the number of collections that contain a subset of samples with very high or very low expression of the gene, respectively (outlier analysis). In parentheses, the number of colorectal cancer collections relative to the previously mentioned results are shown. **B.** Graphical representation of the *PTGS2* and *PTGES* mRNA relative levels (as analyzed in the Oncomine database) dispersion, linear regression, and Pearson correlation coefficient. **C.** Graphical representation of the *PTGS2* and *PTGES* ΔCt (Ct_PTGS2 or PTGES_ – Ct_HPRT_) dispersion, linear regression, and Pearson correlation coefficient, as calculated after qRT-PCR on RNA samples from the cell lines shown.

To study the implication of the COX-prostaglandin(s)-PG receptor(s) genes in colorectal cancer, we collected information on their mRNA expression levels using the OncoMine database (http://www.oncomine.org; Compendia Bioscience, LifeTechnologies). These analyses showed that several members of this pathway are up-regulated in different human tumors (a summary is shown in Fig. 1A). Regarding colorectal tumors *PTGS2*, *PTGES* and *PTGER3* were found up-regulated in many datasets. *PTGS2*, the gene that encodes for COX2 was significantly (*p* < 0.0001) up-regulated in 3 distinct datasets comparing tumor to normal tissue. Moreover, it was expressed at high levels in a subset of the tumor samples of the 21% of the collections (outlier analysis, as defined by Oncomine, expression profile analysis where high or low gene expression is seen in a fraction of samples of the total population/tumor collection without affecting significantly the average value of the population, but receives a high score from Oncomine's algorithm). *PTGES* on the other hand, which encodes mPGES1, was found significantly increased in 3 analyses of colorectal cancer vs. normal tissue, as well as in 8 other cancer type datasets. After outlier analysis high expression was found in samples of the 13% of the collections. *PTGS2* and *PTGES* are both up-regulated in colorectal tumors when compared to normal tissue, while the PG receptor genes expression levels vary considerably, not correlating with the expression of *PTGS2* and *PTGES* (not shown). We then analyzed whether the *PTGS2* and *PTGES* are co-expressed/co-induced in cancer cells calculating the Pearson correlation coefficient of their gene expression values for each tumor sample. There is a very strong correlation between the two genes expression levels in two independent collections of colorectal tumor specimens, Skrzypczak Colorectal 2 and Gaedcke Colorectal, 0.836 and 0.714, respectively (Fig. 1B). Similar results were obtained when we analyzed the data generated by the TCGA Research Network [29] (http://cancergenome.nih.gov/) using the cBioportal for Cancer Genomics [30] (http://www.cbioportal.org/): *PTGS2* and *PTGES* show a tendency towards co-occurrence (*p* < 0.001). On the other hand, cell-line gene expression analysis, using the Cancer Cell Line Encyclopedia [31] tools (http://www.broadinstitute.org/ccle/home) also resulted in a strong correlation of the expression levels of the two genes (Pearson's correlation = 0.45). All these *in silico* findings strongly support the coordinated involvement of the *PTGS2*-*PTGES* axis in colorectal cancer development. Moreover, we analyzed the mRNA levels of various cell lines to find a similar correlation between the levels of the two genes (Fig. 1C).

### mPGES1 and COX2 are co-expressed in human colorectal tumor biopsies

**Figure 2:**
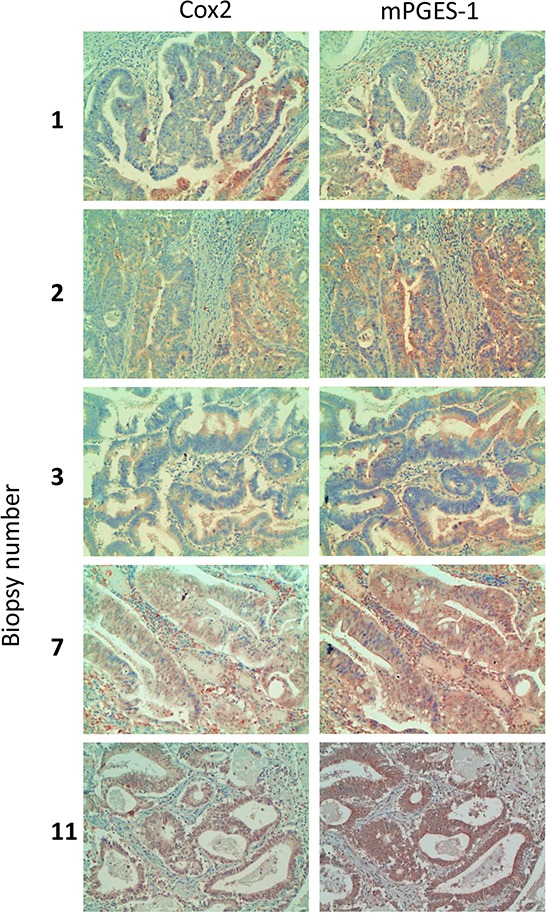
COX2 and mPGES1 expression in human colorectal tumor biopsies Samples from colorectal tumors were processed for immunohistochemistry using antibodies specific for COX2 and mPGES1. Magnification 200 ×.

To confirm if COX2 and mPGES1 are co-expressed in human tumors, we tested by immunohistochemistry their expression in human colorectal tumor biopsies with different location, differentiation state, mucin expression and clinical stage. COX2 was expressed in most of the tumors tested, and almost always accompanied by mPGES1 expression (Table 1 and Fig. 2). Thus, COX2 and mPGES1 are co-expressed in human colorectal tumors as predicted *in silico* and found in colorectal cancer cell lines.

**Table 1:** Expression of mPGES1 and COX2 in human tumor samples and clinical characteristics of the human tumors analyzed (Location, Differentiation state Mucin expression and clinical stage)

BIOPSY NUMBER	Location	Differentiation state	Mucinous	Stage	COX2 staining	mPGES-1 staining
**1**	Rectum/sigmoid	moderate	no	PT3N2M0	++	++
**2**	Cecum	well	no	T3N1Mx	+	++
**3**	Rectum	well	no	NA	++	++
**4**	Rectum	well	no	uT3uN2M1	-	+++
**5**	Ascending	well	yes	T3N2M1	++	++
**6**	Rectum/sigmoid	well	yes	PT3N2Mx	+	+
**7**	Cecum	well	no	PT3N2bMx	++	++
**8**	Transverse	moderate	no	Pt3n0m0	Immune cells. No tumor cells	++
**9**	Transverse/right	well	no	PT4N0Mx	++	+++
**10**	Transverse	moderate	no	PT3N0M0	+	++
**11**	Transverse	moderate	no	PT3N0M0	++	+++

### COX2 overexpression in colon adenocarcinoma cells results in PGs production and mPGES1 increase

**Figure 3:**
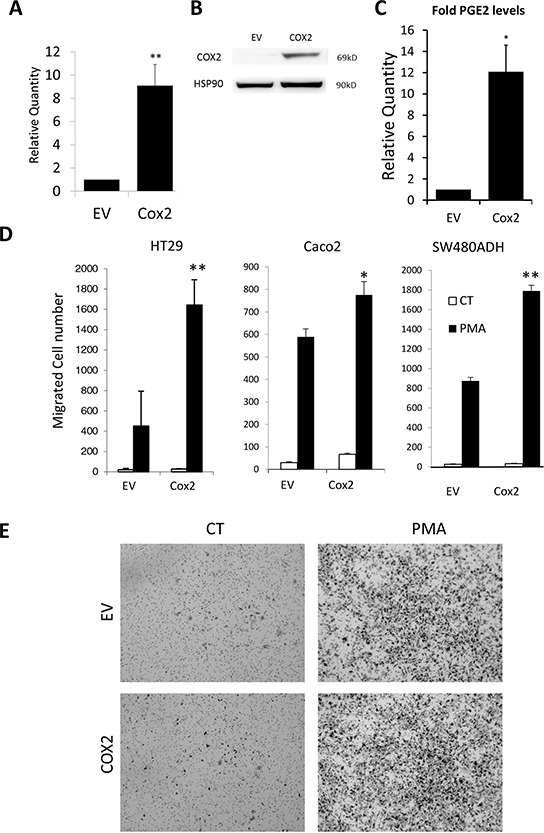
COX2 overexpression enhances cell migration **A.** Relative levels of *PTGES* mRNA in the EV and COX2 HT29 cells as estimated by qRT-PCR. **B.** WBs for COX2 and HSP90 protein levels of the HT29-LucD6-EV and COX2. **C.** Relative PGE_2_ levels of the HT29-EV and COX2 cells supernatants after ELISA quantification. **D.** Cell counts of Boyden chamber migration assays towards serum of the indicated cells stimulated or not with PMA. **E.** Representative images are shown. Magnification 50 ×.

To address the mechanism mediating this coordinate expression, as well as its biological significance, we tested whether overexpression of COX2 enhances tumor progression and if this correlates with mPGES1 up-regulation. For this, we generated several colon carcinoma cell lines, namely SW480ADH, Caco2, SW620, HT29 and HT29-lucD6, stably expressing COX2 and compared them with control cells carrying the empty vector (EV), to avoid any possible artifact attributable to a single cell line. As expected, all COX2-overexpressing cell line expressed more *PTGS2* mRNA, and COX2 protein (not shown). For example, HT29-COX2 express 9 fold more *PTGS2* mRNA than the EV cells (Fig. 3A) and in HT29-lucD6-COX2, COX2 protein levels were higher than HT29-lucD6-EV control cells (Fig. 3B). This overexpression of COX2 resulted in much higher release (1.2 pg/ml/μg of protein for EV cells vs 14.1 pg/ml/μg of protein for COX2-overexpressing HT29 cells) of PGE_2_ released to the supernatant (Fig. 3C) demonstrating that overexpressed COX2 is functional.

### COX2-overexpression confers an enhanced pro-tumorigenic phenotype

PG production is associated to higher migration of colon tumor cells [25]. Thus, we next evaluated the effect of COX2 overexpression on cell migration. For this, Boyden chamber assays were performed for the HT29, Caco2 and SW480ADH -EV and -COX2 cell lines. In all cases COX2-overexpressing cells showed a significant tendency (*p* < 0.05, *n* = 3 for each cell line) to migrate more than the EV cells. For the HT29 cell line, an average of 20 ± 3 EV and 29 ± 4 COX2-overexpressing cells migrated in 48 h. This comparison resulted in 28 ± 4 EV and 69 ± 6 COX2-overexpressing Caco2 cells and 24 ± 3 EV and 33 ± 5 COX2-overexpressing SW480ADH cells. Migration was strongly increased when cells were pretreated with PMA Interestingly, in all the cases, COX2-overexpressing cells migrated significantly more than their EV counterparts (Fig. 3D–3E). PMA treatment did not affect COX2 protein levels (not shown).

**Figure 4:**
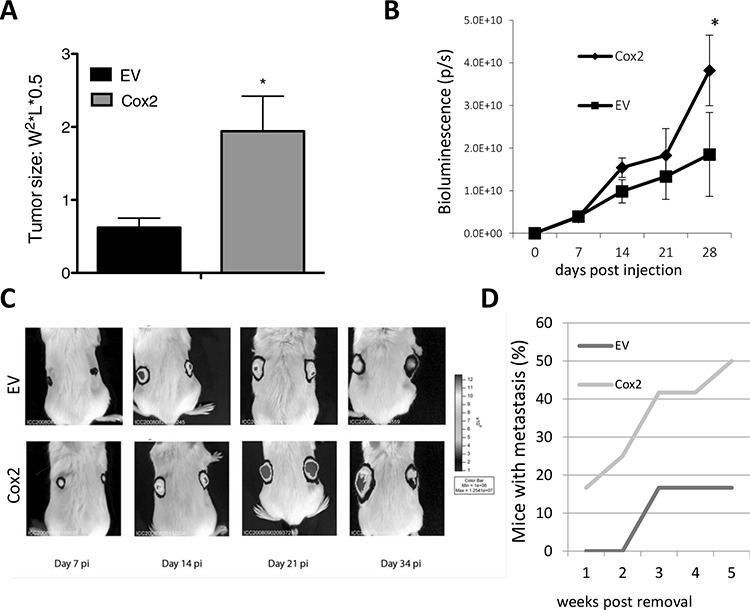
COX2 overexpression increases tumor growth capacity and metastasis of HT29 cells in nude and SCID mice **A.** Growth of HT29-EV and HT29-COX2 subcutaneous tumors in nude mice (10 mice per group). Tumor size (±SEM) 5 weeks post-injection. **B.** Growth of HT29-lucD6-EV and -COX2 subcutaneous tumors in SCID mice (5 mice each). Growth was evaluated by light emission of the cells after D-Luciferin injection. **C.** Representative images of animals carrying the mentioned tumors, pseudocolored according to bioluminescence intensity. **D.** Percentage of mice with metastasis detected by bioluminescence after removal of the primary tumor.

We next sought to test if COX2 overexpression favored tumor growth *in vivo*. HT29-COX2 cells produced larger tumors than the EV cells in the nude mice xenograft cancer progression model (Fig. 4A). Similar experiments were performed in SCID mice using the HT29-lucD6 derivatives and evolution of the tumors was monitored by bioluminescence. Again, COX2-overexpressing cells grew faster than the EV cells (Fig. 4B and 4C). These results indicate that COX2 overexpression promoted faster tumor growth *in vivo*.

Moreover, we were able to detect metastatic tumors 21 days after the surgical removal of primary HT29-lucD6-EV cells derived tumor in a minor fraction of the mice (17%). On the contrary, in mice inoculated with HT29-lucD6-COX2 cells we detected metastasis were detected earlier and 5 weeks post inoculation half of the animals had metastasis (Fig. 4D). All metastases occurred in the lung and were always found near a blood vessel (not shown). This supports the role of COX2 in favoring lung metastasis as it has been reported for mammary tumors [5, 32, 33].

### COX2 overexpression induces PTGES through EGR1

**Figure 5:**
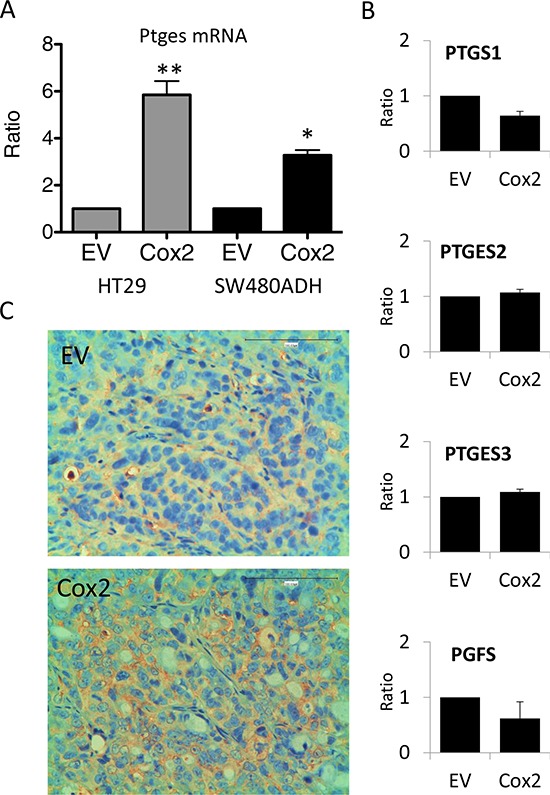
COX2 overexpression induces mPGES1 up-regulation **A.** *PTGES* mRNA levels are higher in HT29-LucD6 or SW480ADH cells overexpressing COX2 comparing to EV cells, as estimated by qRT-PCR. **B.** mRNA levels of different genes encoding for key enzymes of the prostanoid biosynthesis pathway in HT29-EV and COX2 cells. No significant differences were found. **C.** Immunohistochemistry for mPGES1 on tumor samples generated after subcutaneous injection of the cells, showed that COX2 tumors express 2–3 fold more mPGES1 than the EV ones. Magnification 200 ×.

**Figure 6:**
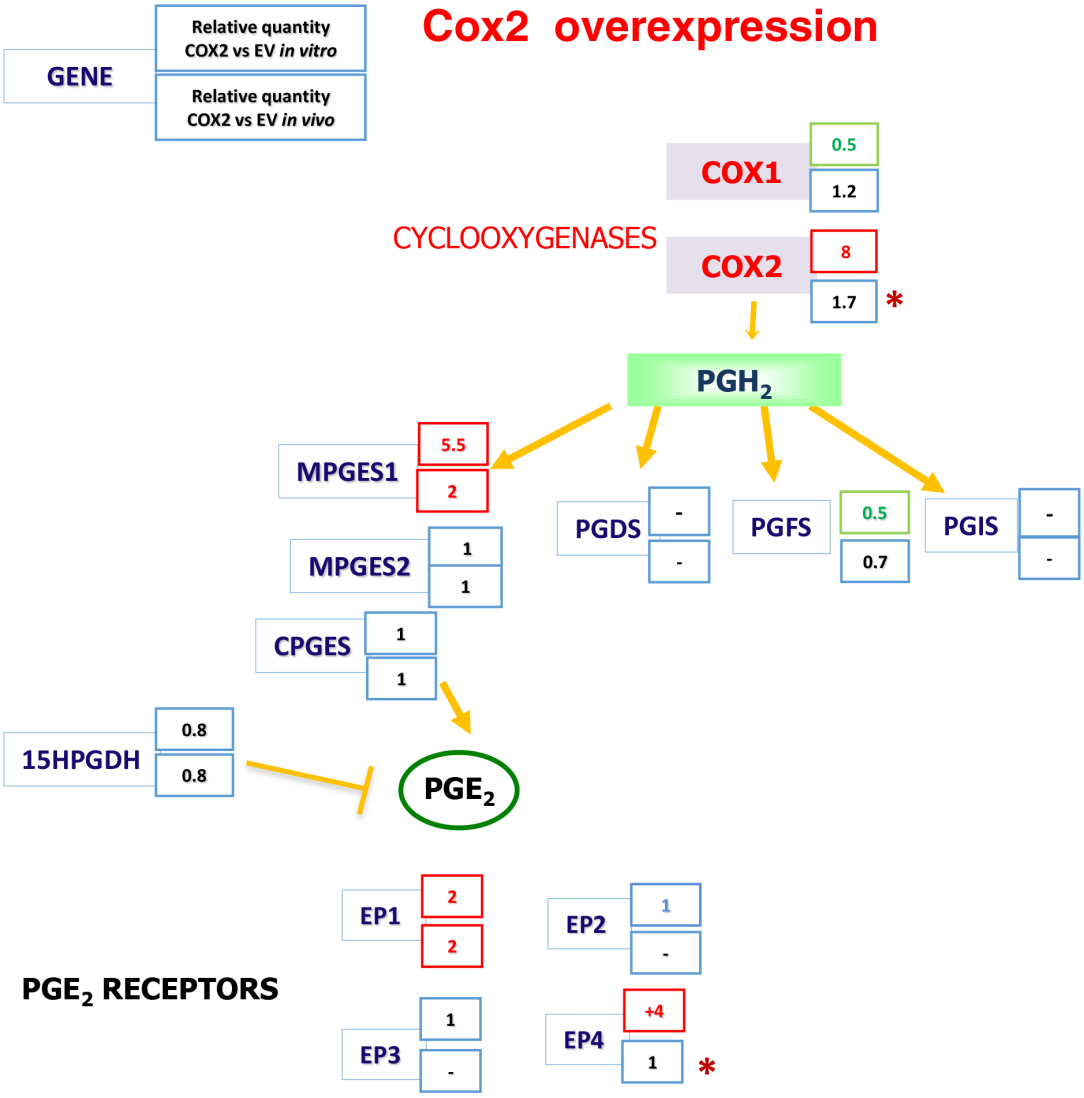
The Cyclooxygenases-PGE_2_ pathway gene expression analysis after COX2 overexpression Expression of PG pathway genes *in vitro*, in cells in culture, *in vivo*, in the tumors extracted from nude mice. Summary from the gene expression data obtained by qRT-PCR for each of the genes of the pathway analyzed, averages of three independent experiments in culture and for the tumors of 4 mice are shown. -: undetectable. *the mRNA levels of these genes were found induced in the EV cells derived tumors as compared to the levels of the EV cells in culture.

Interestingly, we found that mPGES1 was significantly up-regulated in all COX2-overexpressing colorectal carcinoma cell lines tested (an example of HT29 and SW480ADH is shown in Fig. 5A and for Caco2-COX2 a 14 ± 3 fold increase of PTGES was observed when comparing to EV cells). This gene induction by COX2 overexpression is specific to *PTGES* since the levels of other components of the PG pathway, such as *PTGES2*, *PTGES3* or *PGFS* remained unchanged (Fig. 5B). More interestingly, *in vivo* subcutaneous tumors in nude mice express higher levels of mPGES1 when they originate from COX2-overexpressing cells (Fig. 5C). Additionally, we analyzed mRNA levels of several components of the COX-PG synthases-PG receptors pathway both *in vitro*, in cells in culture, as *in vivo*, in the tumors extracted from the nude mice to find a 2-fold increase of PTGES levels in the COX2 tumors (Fig. 6). These data confirmed that COX2 overexpression in colon carcinoma cells concomitantly leads to the up-regulation of mPGES1-PGE_2_ pathway. Interestingly, *PTGS2* expression was increased in control HT29-EV xenotransplanted cells derived tumors as compared to the levels of these cells in culture. This may explain the lower differences between the lines found *in vivo*.

**Figure 7:**
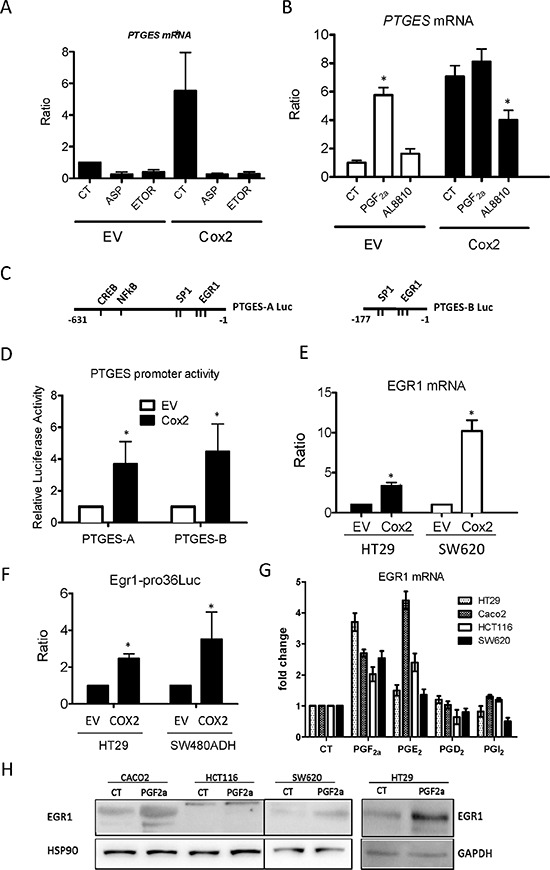
Regulation of mPGES1 expression by *COX2* and *EGR1* **A.** mPGES1 expression is reduced by aspirin (ASP, 1 μM) or etoricoxib (ETOR, 1 μM) treatment for 24 h, in both HT29-LucD6 EV and COX2 cells as quantified by qRT-PCR. **B.** *PTGES* mRNA is increased by PGF_2α_ (1 μM) in EV cells while it is reduced by AL8810 (10 μM) treatment (24 h) in HT29-LucD6-COX2. **C, D.** Relative luciferase activity of cells transfected with the “full length” (PTGES-A, -631 to -1) and “proximal” (PTGES-B, -177 to-1) constructs of the human *PTGES* promoter transfected in HT29-EV and –COX2 cells. **E.** mRNA levels of *EGR1* were quantified by RT-PCR in samples from EV and COX2-overexpressing HT29-LucD6 and SW620 cells. **F.** Relative luciferase activity of an EGR1 reporter construct EV and COX2 in HT29 and SW480ADH cells. EGR1 mRNA **G.** and protein **H.** induction after 24 h treatment of the indicated cell lines with 1 μM of the PGs shown. *p < 0,005

Next, we studied the molecular mechanisms responsible for mPGES1 up-regulation. Inhibition of COX2 enzymatic activity with Aspirin or Etoricoxib reverted the induction of mPGES1- in COX2-overexpressing cells (Fig. 7A), confirming that it was due to enhanced COX2 activity in those cells. We then examined the COX2-derived prostanoid(s) involved in the up-regulation of mPGES1. PGE_2_ treatment of HT29 cells did not affect *PTGES* mRNA levels (not shown) while PGF_2α_ caused a 6-fold increase (Fig. 7B). Besides, treatment with PGF_2α_ receptor (FP) antagonist AL8810 significantly reduced the *PTGES* mRNA induction in COX2-overexpressing cells, indicating a COX2/PGF_2α_ dependent induction. Similar results were obtained in SW480ADH cells (not shown). To identify the signaling pathways involved, we transfected the EV and the COX2 HT29 cells with luciferase reporter constructs containing the “full length” *PTGES* promoter, construct A (−631 to − 1 bp), or just the proximal promoter region, construct B (−177 to − 1 bp) [27] as shown in Fig. 7C. Luciferase activity was high when any of the two constructs were transfected in the COX2-overexpressing cells (Fig. 7D). This confirms that COX2 activates the *PTGES* promoter and indicates that the transcription factor binding sites responsible for this *PTGES* transcriptional up-regulation are localized in the proximal promoter region, where three *EGR1* response elements have been identified [19]. Similar results were obtained with SW480ADH and Caco2 cells (not shown). In agreement with these results, we found that *EGR1* mRNA is up-regulated in COX2-overexpressing cells and that an *EGR1*-reporter construct was more active in COX2-overexpressing cells (Fig. 7E and 7F). Similar results were obtained in Caco2 cells (not shown). Conversely, PGF_2α_ treatment increased 2-fold this reporter construct activity in SW480ADH (±0.1) and HT29 (2.2 ± 0.3) cells. Additionally, we tested what PGs were able to induce EGR1 in different colorectal cancer cell lines (Fig. 7G for mRNA and 7H for protein). Finally, EGR1 overexpression caused a great increase in *PTGES* mRNA in HT29 (14.26 ± 2.15, *p* < 0.01) and Caco2 cells (8.3 ± 2.9, *p* < 0.05), supporting the direct regulation of *PTGES* expression by EGR1.

**Figure 8:**
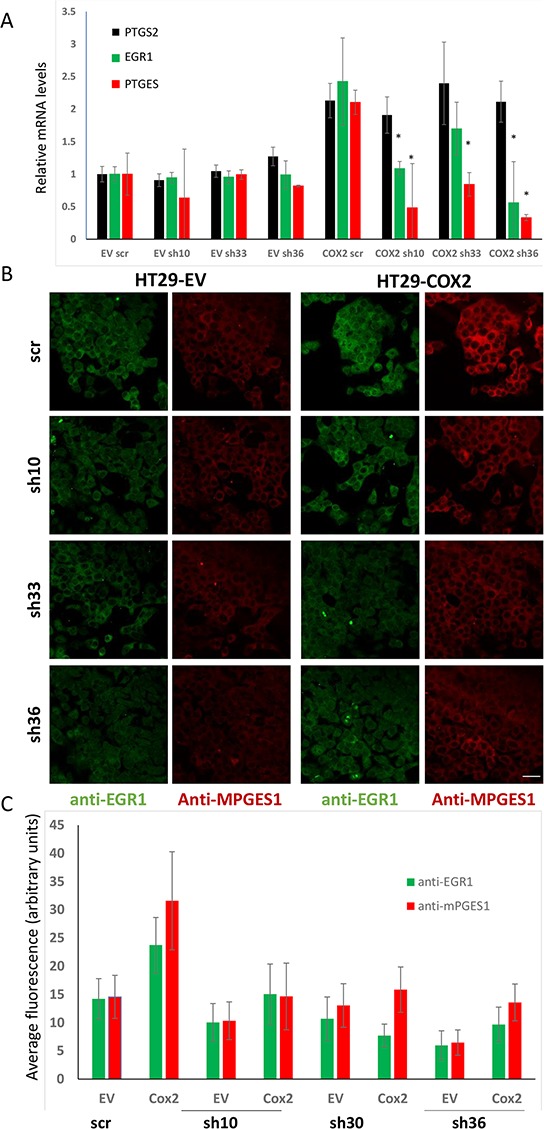
EGR1 silencing reduces the mPGES1 up-regulation by COX2 **A.** Relative levels of *PTGS2*, *EGR1* and *PTGES* mRNA, estimated by RT-PCR analysis of HT29-EV and –COX2 cells transfected with 3 different shRNA expressing plasmids, specific for EGR1 or one shRNA without any known mammalian target (scr). **B.** Immunofluorescence for mPGES1 and EGR1 of HT29-EV and –COX2 cells transfected as mentioned above. **C.** Fluorescence quantification averages of the images of three independed experiments, as in **(B)** *p < 0,001. Bar: 32 μm.

To further confirm this, we tested whether RNA interference for *EGR1* would reduce the expression levels of *PTGES.* As it can be observed in Fig. 8 (upper panel), *EGR1* knockdown reduced the *PTGES* mRNA levels in COX2-overexpressing HT29 cells without affecting *PTGS2* levels (similar results were obtained in SW480ADH cells, not shown). Besides, EGR1 knockdown also reduced mPGES1 protein levels induced by COX2 as shown by immunofluorescence (Fig. 8, lower panels and graph). Although EGR1 knockdown does not significantly affect neither EGR1 nor mPGES1 levels in EV cells, probably due to their already low EGR1 levels, it prevents the up-regulation of EGR1 and mPGES1 in COX2-overexpressing cells. This was observed both at the mRNA and protein level. Furthermore, strong correlations of *PTGS2* or *PTGES* and *EGR1* mRNA levels in several cancer datasets were found, and particularly in Skrzypczak Colorectal 2 dataset, Pearson's correlation was 0.84 for *PTGS2* and *EGR1* and 0.647 for *PTGES* and *EGR1* mRNA levels (Table 2). Altogether these results demonstrate that mPGES1 is induced by COX2 overexpression, via autocrine PGs release, likely PGF_2α_, through an *EGR1*-dependent mechanism.

**Table 2:** Pearson's correlation coefficient calculated from the gene expression data for the genes shown in the two colorectal tumor collections (Gaedcke colorectal and Skrzypczak Colorectal 2; Oncomine)

	PTGES vs PTGS2	PTGES vs EGR1	PTGS2 vs EGR1
Skrzypczak Colorectal 2	0.836	0.647	0.840
Gaedcke Colorectal	0.714	0.408	0.235

### mPGES1 overexpression promotes tumor growth *in vivo*

**Figure 9:**
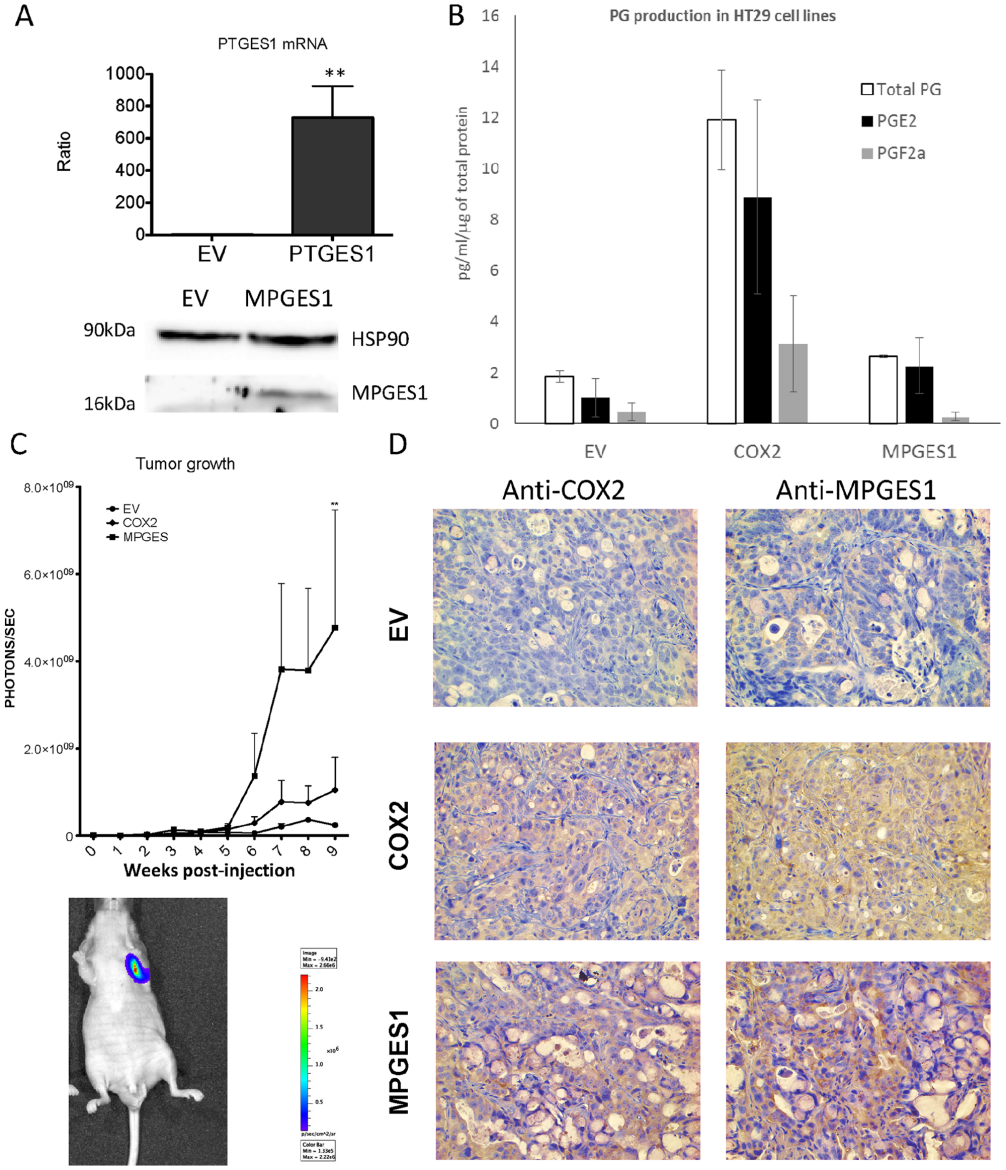
mPGES1 overexpression in colon cancer cells **A.** *UP*: mRNA levels of *PTGES* in control or mPGES1 overexpressing cells as estimated by qRT-PCR. *DOWN*: WB against mPGES1 and HSP90 in samples of the above mentioned cells. **B.** PGE_2_, PGF_2α_ and total PG levels in supernatants of EV, COX2 and mPGES1 transduced HT29-Luc-D6 cells, quantified by ELISA immunoassay. The graph shows means and standard error of three independent experiments. **C.** Quantification of tumor produced bioluminescence of HT29-LucD6-EV, -COX2 and –mPGES1 cells inoculated subcutaneously in *nude* mice (5 per group). One *Nude* mouse had developed metastasis before week 9 post injection in the axillar lymph node (shown in the lower part). **D.** At the end of the experiment mice were sacrificed, tumors were extracted and processed for immunohistochemistry using antibodies specific for COX2 and mPGES1. Magnification 200 ×.

The above results suggested that mPGES1 induction is a key point in the pro-tumorigenic activity of COX2. To confirm this we generated a HT29-lucD6 cell line carrying the *PTGES* gene, expressing more than 500-fold higher mRNA levels and confirmed the overexpression of the protein (Fig. 9A). mPGES1 overexpression was not as effective as COX2 to increase the total PG levels but increased the proportion of PGE_2_ in the total PGs while it reduced the proportion of PGF_2α_ (Fig. 9B).

We then compared the *in vivo* tumorigenic activity of both mPGES1 and COX2 expressing lines in mouse xenograft experiments. As shown in Fig. 9C, COX2 tumors grew faster than the EV ones, as expected. Interestingly, the mPGES1-overexpressing cells formed tumors much faster that grew larger than the other two groups, with much higher bioluminescence signal, indicating higher numbers of living cells. Histological observations of the tumors showed more necrotic regions in the ones derived from the control cell line. Remarkably, the mPGES1 tumors were less compact, with less stroma, composed mainly of mucinous cells indicating higher degree of cell differentiation into the tumor. Immunohistochemical staining of the sections with an mPGES1 antibody showed stronger staining in HT29-COX2 derived tumors than in the HT29-EV generated tumors and as expected very strong staining in the HT29-mPGES1 tumors (Fig. 9D). This staining was specifically localized in the tumor cells, while no staining was observed for other cell types like immunologic infiltrate or stroma.

## DISCUSSION

Epidemiological studies as well as experimental models have long suggested that COX2 plays an important role in colorectal tumorigenesis [6, 7, 34–36]. However, despite the wealth of data on COX2 and cancer, very few direct evidence of COX2 overexpression in colon carcinoma cells with tumor properties *in vivo* have been published. More importantly, no studies have comprehensively addressed the downstream PGs/PG receptors pathways involved. Several studies have attributed COX2 effects to PGE_2_. Not withstanding this, the 2 published reports on the effect of genetic deletion of mPGES1 in mice on colon cancer are contradictory indicating that the mPGES1 deficient mice have either reduced [37] or accelerated [38] intestinal tumorigenesis, probably due to differences in the inflammatory responses.

Thus, we studied the possible downstream COX2 effectors, as PG synthases-PG receptors, both in human colorectal cancer samples and in animal models. Data analysis from numerous collections of human tumors indicated that *PTGS2* and *PTGES* expression correlated strongly, while for the rest of the members of the mentioned pathway we did not find any correlation. Importantly, we confirmed the *in silico* results in human colorectal tumor biopsies, being the first evidence of a relationship between COX2 activity and mPGES1 expression levels in the same tumor settings.

To address the mechanism and consequences of this association, we overexpressed COX2 in several human carcinoma cell lines. Our results agree with previous work from our group and other groups, which have reported enhanced cell migration in cells with spontaneous up-regulation of COX2 and in cells that overexpress COX2 [25, 39–42]. Besides, we report for the first time that COX2 overexpression alone increased tumor growth and the number of metastases in mice, confirming that the overexpression of COX2 plays an important role in colon cancer progression and dissemination.

The tumorigenic effects of COX2 are considered to be due to the production of PGs [43], particularly PGE_2_ [44, 45]. The PGE_2_ synthase mPGES1, has been associated with carcinogenesis and proposed as marker of poor prognosis [13, 14, 46]. Both *PTGS2* and *PTGES* genes share a similar transcriptional regulation by certain growth factors and pro-inflammatory stimuli [28, 47, 48]. However, the possible link between these enzymes in colon cancer was not addressed before.

Interestingly, we found that COX2 overexpression caused the up-regulation of mPGES1 thus leading to increased PGE_2_ and PGF_2α_ synthesis. We were able to demonstrate that this up-regulation depended directly on COX2 activity using specific and non-specific COX2 inhibitors. Interestingly, mPGES1 induction depends, at least partially, on PGF_2α_, since an FP antagonist, AL8810, could revert it, giving a previously unrecognized role of this PG/FP in colon cancer. AL8810 is a PGF_2α_ receptor antagonist that would only avoid activation of the receptor by newly synthetized PGF_2α_. However, this compound has itself a low level intrinsic capacity to activate the receptor, thus allowing low level induction of EGR1 and mPGES1. On the other hand, PGE_2_ is also able to induce EGR1 in certain circumstances and cell lines, thus the contribution of both PGs to the observed effect cannot be discarded. In this regard, it is well established that both PGF_2α_ and PGE_2_ can induce EGR1 [16, 17]. Further studies are necessary to evaluate the effect of PGF_2α_ on tumor progression and metastasis.

Using the PTGES promoter reporter constructs we confirmed the EGR1 and discarded NF-kB or CREB activation since the binding sites identified for these factors are present in the “full length” but not in the “proximal promoter” construct. EGR1, a transcription factor known to be important for the transcriptional regulation of *PTGES* in other cell types [19, 28] is responsible for the induction of mPGES1 by COX2. More importantly, silencing *EGR1* gene with RNA interference abrogated COX2-mediated *PTGES* induction proving a direct implication of *EGR1* in this process. Supporting these results, data obtained from *in silico* analysis of tumor microarray gene expression levels show a strong correlation between the expression levels of the three genes, in colorectal tumors. Our results are also in agreement with reports of EGR1 down-regulation by NSAIDs [27, 49] and up-regulation by PGE_2_ [50]. Besides, there are some reports implicating *EGR1* in colorectal and gastric cancer cell proliferation and metastasis [51, 52]. Moreover, *EGR1* has been proposed to be one of the genes with increased expression early in the development of colorectal cancer [53] and to be implicated in stem cell marker regulation [54].

We also found that mPGES1-overexpressing colon carcinoma lines grow tumors faster, even faster than the COX2-overexpressing ones, indicating that mPGES1 activity alone would be sufficient to promote tumor growth. *In vitro*, mPGES1 overexpression increases PGE_2_ levels by taking advantage of the basal PGH_2_ production, conversely reducing the levels of other PGs like PGF_2α_. The change of the balance between the different PGs towards PGE_2_ may be enough to stimulate faster tumor growth.

In conclusion, we found here that COX2 overexpression confers a more aggressive tumor phenotype in colon carcinoma cells and that this phenotype can be mimicked by mPGES1 overexpression. Besides, COX2 overexpression was functionally and casually linked to mPGES1 in human tumor samples and in all cell lines tested *in vitro* and *in vivo* derived tumors. Finally, our results unraveled a new molecular mechanism for this association involving COX2*PGF2a*EGR1*mPGES1 that likely makes the sustained expression of COX2 the principal event necessary for tumor promotion by this cascade.

## MATERIALS AND METHODS

### Cell lines

The Caco-2, HCT116, SW620 and HT29 cell lines were obtained from the ATCC (LGC Standards, Barcelona, Spain). HT29-lucD6, stably expressing Firefly Luciferase, obtained from Caliper Life Sciences. The SW480ADH and SW480R cells [23] were a kind gift from Dr. Alberto Muñoz, Madrid, Spain. The Caco2 and HCT116 cells were obtained from the *Centro de Investigaciones Biológicas* Tissue culture Repository (Madrid, Spain). Cell lines were validated with the StemElite ID system (Promega). Cells were grown as described [24, 25].

The COX2-overexpressing cell lines and the empty vector controls (EV) were generated by transfection with the pBABE-puro vector carrying or not the human COX2 gene, using Lipofectamine 2000 (Life Technologies) according to the instructions of the manufacturer. Cells were selected for one week with 2 μg/ml puromycin and then several independent mass cultures were obtained. COX2- and mPGES1-overexpressing cell lines were also generated by transduction with lentiviral particles carrying the mentioned genes or the empty vector, after subcloning to the pSMPUW-IRES-Bsd (Cell Biolabs) following the manufacturer's instructions. Cells were grown under blasticidin (Invivogen) selection (4 μg/ml).

Aspirin and phorbol myristate acetate (PMA) were acquired from Sigma-Aldrich. Etoricoxib, from Merck Sharp & Dohme. AL8810, PGE_2_ and PGF_2α_ were from Cayman Chemicals.

### Tumor growth in immunodeficient mice

We used three strains of immunodeficient mice, Swiss Nude (Crl:NU(Ico)-Foxn1^nu^, Charles River Laboratory) and SCID (BALB/cJHan Hsd-Prkdcscid) or NOD-SCID (NOD.CB17-Prkdcscid/NCrHsd) from Harlan Laboratory. In the experiments with nude and NOD-SCID mice, groups of 4–6 animals were injected with 0.5× or 3 × 10^6^ cells from generated cell lines. For the SCID mice experiments, groups of 10 animals were injected with 3 × 10^6^ HT29-lucD6-COX2 or HT29-lucD6-EV cells in each flank of the back. Tumor volume was estimated with calipers by the following calculation: ([width]^2^ × [length])/2. For HT29-LucD6 cell lines, bioluminescence acquisition on anesthetized mice (isofluorane gas, 1.5%, Abbott, Madrid, Spain) was performed using an IVIS Lumina II (Caliper Life Sciences) after intraperitoneal injection of 150 mg/kg of body weight of D-Luciferin (Promega). The luminescent signal was quantified with Living Image 3.2 software and expressed as photons/s (Average radiance). The animal experimentation complied with National and European Union legislation and was supervised by the center's Ethics Committee.

### Histological analysis and immunohistochemistry

Tumors from mice were fixed in 4% phosphate-buffered formalin (pH 7.4), and 3 μm paraffin-embedded sections were stained with hematoxylin-eosin or immunostained as described previously [26]. Antibodies used for immunohistochemistry: anti-COX2 (Sigma-Aldrich) or anti-mPGES1 (Cayman Chemicals Europe) and secondary antibody conjugated with HRP (Envision + Dual link System HRP, Dako). Finally, sections were developed using DAB solution (Liquid DAB + substrate chromogen system, DAKO K3468), counterstained with hematoxylin and images were taken with a LEICA DMD108 Digital Microimaging Device (Leica Microsystems). Human tumor biopsies were obtained after the approval of the Ramon y Cajal University Hospital Ethics Committee according to Spanish and EU laws.

### *In vitro* cell migration assay

Migration assays were carried out basically as described [25]. Briefly, 2 × 10^4^ cells were placed in 8 μm pore size transwell filter chambers in MEM without serum, prior 0.5 h treatment with PMA, 100 nM. The inserts were placed in wells filled with MEM 20% FBS. After 48 h at 37°C, they were processed and quantified as described [25].

### mRNA extraction and quantitative real time PCR

Total RNA was prepared from colon carcinoma cell lines by the TRIzol reagent (Invitrogen, Alcobendas, Spain) according to the manufacturer's instructions. Total RNA was reverse transcribed into cDNA and the quantitative PCR was performed using the GoTaq 2-Step RT-PCR system (Promega). Relative mRNA levels to the housekeeping *HPRT* gene and to the experimental control point were calculated using the 2^−ΔΔCT^ formula from the values obtained. A list of the gene specific primers used can be found in Table 3.

**Table 3:** Oligonucleotide primers used in this study

Gene	Primer Sequence
*HPRT*	CTGGAAAGAATGTCTTGATTGTGG
	CATCTTTGGATTATACTGCCTGAC
*PTGS1*	GAAACCCTACACCTCCTTCC
	GCATCAATGTCTCCATACAATTCC
*PTGS2*	CGAGGTGTATGTATGAGTGTG
	GTGTTTGGAGTGGGTTTCAG
*PTGES*	CTGGTCATCAAGATGTACGTG
	GGGTAGATGGTCTCCATGTC
*PTGES2*	TCGCAACAACTAAATGACTCC
	CTGGGTAGTAGGTGATGATCTC
*PTGES3*	AGAAAGGGCAAAGCTTAATTGG
	ATCATCTGCTCCATCTACTTCTG
*HPGD*	GCAGTTTGAACCTCAGAAGAC
	CACTCCAGCATTATTGACCA
*PHDS*	GCATGACGGAACAATAGGAC
	GAACAGAGCAGAGACATCCA
*HPGDS*	GGGAGAGCAGAAATTATTCGT
	AGAGTAAGTCCATCAACTTCCA
*PGFS (AKR1C3)*	TTCTCCAATGTCTCTAAAGCCA
	ATCCTGCATCCTTACACTTCTC
*PGIS*	AGAAATCTACACAGACCCAGAG
	TGTAATTCTTCAGCCGTTTCC
*PTGER1*	GTCGGTATCATGGTGGTGTC
	CGCAGTAGGATGTACACCCA
*PTGER2*	GTCTGCTCCTTGCCTTTCAC
	TGAACGCATTAGTCTCAGAACAG
*PTGER3*	TCAACCTTGATGTGGAGCGA
	GCAAATTCAGGGAAGCAGGA
*PTGER4*	TCTTACTCATTGCCACCTCCC
	GTTGACGAATACTCGCACCAC

### Prostaglandin quantification

PGE_2_, PGF_2α_ and total PGs were quantified using ELISA kits from Cayman Chemicals Europe, following the manufacturer's instructions. The quantifications were performed on serum free medium incubated with cells during 4 hours at 37°C.

### Luciferase reporter assays

Cells were transfected with luciferase reporter constructs, namely PTGES-A-Luc, PTGES-B-Luc [27] or *EGR1*-pro36-Luc [28] together with the SV40-Renilla plasmid at a 80:1 proportion, with Lipofectamine 2000 (Invitrogen) according to the manufacturer's instructions. 6 h after transfection, media were replaced and 48 h later cells were lysed and processed using the Dual Luciferase Assay kit (Promega) and bioluminescence was measured in a Sirius manual luminometer (Berthold Detection Systems).

### RNA interference

For stable RNA interference, the following Mission shRNA lentiviral vector constructs (Sigma Aldrich) were used: TRCN0000273910, TRCN0000013836, TRCN0000013833. Viruses were produced and cells were transduced following the manufacturer's instructions. For transient knockdown, these constructs were transfected with Metafectene (Biontex Laboratories, GmbH) following the manufacturer's instructions. Knowdown efficiency was evaluated by RT-PCR and immunofluorescence.

### Western blot analysis

Western blots (WB) were carried out as described previously [24]. Membranes were incubated with specific antibodies against COX2, mPGES1 (Cayman Chemicals Europe) or anti-*EGR1* (sc-189) or anti-HSP90 (sc-7947) antibody (Santa Cruz Biotechnology).

### Immunofluorescence

Cells were plated on 12 mm diameter cristal coverslips the day after transfection. 48 h later cells were washed with PBS and fixed with 4% paraformaldehyde in PBS for 20 min. Cells were permeabilized with 0.1% Triton X-100 for 20 min and blocked with 2% BSA in PBS for 1 h. Antibody incubations were done in 1% BSA in PBS. Anti-mouse and anti-rabbit secondary antibodies (Alexa 488 and 555) were from Life Technologies. Images were acquires with a LSM510 laser confocal microscope (Zeiss) all in the same conditions and fluorescence intensity was calculated with the Fiji software (adapted version of Image J).

### Statistical analysis

Results are expressed as means ± SEM. The Student's t test were used for comparisons. *p < 0.05 and **p < 0.01 denote statistical significance. The statistical analysis was performed using the GraphPad Prism 4.0 statistical software.
